# Socioeconomic inequalities in breast cancer incidence and mortality in Europe—a systematic review and meta-analysis

**DOI:** 10.1093/eurpub/ckw070

**Published:** 2016-05-23

**Authors:** Adam Lundqvist, Emelie Andersson, Ida Ahlberg, Mef Nilbert, Ulf Gerdtham

**Affiliations:** 1 IHE, The Swedish Institute for Health Economics, Lund, Sweden; 2 Institute of Clinical Sciences, Department of Oncology, Lund University, Lund, Sweden; 3 Department of Clinical Sciences, Lund University, Lund, Sweden

## Abstract

**Background:** Breast cancer is the leading cause of female cancer in Europe and is estimated to affect more than one in 10 women. Higher socioeconomic status has been linked to higher incidence but lower case fatality, while the impact on mortality is ambiguous. **Methods:** We performed a systematic literature review and meta-analysis on studies on association between socioeconomic status and breast cancer outcomes in Europe, with a focus on effects of confounding factors. Summary relative risks (SRRs) were calculated. **Results:** The systematic review included 25 articles of which 8 studied incidence, 10 case fatality and 8 mortality. The meta-analysis showed a significantly increased incidence (SRR 1.25, 1.17–1.32), a significantly decreased case fatality (SRR 0.72, 0.63–0.81) and a significantly increased mortality (SRR 1.16, 1.10–1.23) for women with higher socioeconomic status. The association for incidence became insignificant when reproductive factors were included. Case fatality remained significant after controlling for tumour characteristics, treatment factors, comorbidity and lifestyle factors. Mortality remained significant after controlling for reproductive factors. **Conclusion:** Women with higher socioeconomic status show significantly higher breast cancer incidence, which may be explained by reproductive factors, mammography screening, hormone replacement therapy and lifestyle factors. Lower case fatality for women with higher socioeconomic status may be partly explained by differences in tumour characteristics, treatment factors, comorbidity and lifestyle factors. Several factors linked to breast cancer risk and outcome, such as lower screening attendance for women with lower socioeconomic status, are suitable targets for policy intervention aimed at reducing socioeconomic-related inequalities in health outcomes.

## Introduction

Both individual factors and environmental factors contribute to the risk of cancer and the prognosis for affected patients. In cancer epidemiology, the impact from socioeconomic status (SES) on incidence rate and prognosis is increasingly recognised. High SES has been linked to a higher risk for breast cancer and malignant melanoma, whereas low SES is associated with adverse prognosis in for example stomach cancer, lung cancer, prostate cancer and ovarian cancers.^1^^,^^2^ Incidence, case fatality and mortality rates are affected by both illegitimate factors of inequality, ‘circumstances’ and legitimate factors of inequality, ‘effort’. Circumstances are factors exogenous to the person, including age, access to health care and childhood SES, while efforts are factors that can be influenced by the person, including lifestyle factors such as smoking, alcohol use and physical activity.^3^ Policies of equal-opportunity requires an understanding of how circumstance and effort contribute to observed inequality. It is especially hard to identify factors of effort and how these are influenced by circumstance. For instance, it can be argued that smoking attributed to family background is a circumstance and hence an illegitimate factor of inequality.^4^

Breast cancer is the leading cause of female cancer in Europe, is estimated to affect more than one in 10 women and accounts for 28.8% of female cancer.^5^ Individual factors, e.g. ethnicity, family history, age, reproductive factors, alcohol intake, weight, physical activity, hormone therapy and oral contraceptives, has been found to influence the risk of breast cancer.^6^ Tumour stage at diagnosis, access to health care, comorbidity, smoking, BMI, stress and social support have been linked to breast cancer prognosis and risk of death. Individual factors and SES may be associated, which in breast cancer may be exemplified by reproductive factors, hormone therapy, smoking and access to health care in relation to SES. Partly contradictory observations have been reported with higher incidence of breast cancer for women residing in higher socioeconomic areas, whereas no significant correlation has been found between mortality and residential area.^7^

The purpose of this systematic literature review and meta-analysis was to summarise the published literature on the association between SES and breast cancer incidence, case fatality and mortality in European women, with a focus on the effect of other factors, including reproductive factors, mammography screening, tumour characteristics, treatment factors, comorbidity and lifestyle factors.

## Methods

The PRISMA guidelines for systematic reviews were followed.^8^ Before initiating the systematic review several test searches were made in PubMed to find a relevant search strategy that balanced sensitivity and specificity. Elaboration with different combinations of medical index subheadings (MeSH) and title/abstract (Tiab) terms was used to find a suitable search strategy. The final search strategy used is presented in Supplementary table S1 in the Supplementary material.

Eligibility criteria for inclusion in the systematic review were: evaluation of female breast cancer incidence, case fatality or mortality as outcome; use of education, income, occupation or an index including one of these as a measurement of SES; estimates of relative measures of association; use of population-based individual-level data from a European OECD-country; be written in English and be published in the last 10 years. The Newcastle-Ottawa quality assessment scale was used to evaluate risk of bias within the individual studies. The assessment of study quality is based on three perspectives: the selection of the study groups; the comparability of the groups and the ascertainment of either the exposure or outcome of interest. A scoring system has been developed according to these perspectives and studies can be rewarded nine stars at best and zero at worse.^9^ Two researchers (A.L. and I.A.) independently reviewed both titles, abstracts and full-texts. Studies not adhering to the inclusion criteria were excluded. Studies that at least one of the researchers assessed as relevant were included in the next stage of the literature review. In case of doubt, this was resolved through discussion with a third researcher (E.A.).

One author (E.A.) extracted data from the included articles into an electronic database, and one author (A.L.) verified the coded information against the original articles. The study characteristics included the country or region of the study, outcome measure, socioeconomic measures (education, income, occupation or an index), covariates, population, data source, study design and statistical method.

The studies were divided into three sections based on the outcome measure used: incidence, case fatality or mortality.^10^ Incidence measures the risk of being diagnosed with breast cancer in the general population. Case fatality measures the risk of dying in the subset of the population that has been diagnosed with breast cancer. Breast cancer mortality measures the risk of dying of breast cancer in total, i.e. it is the product of incidence and case fatality.

Relative measures of association in the form of hazard ratios [HR], rate ratios [RR], odds ratios [OR] or standardised incidence ratios [SIR] were extracted from the articles. Comparisons were made between the reference category and the category at the other end of the spectra for the socioeconomic measures (e.g. highest compared with lowest income group). If available, more sophisticated measures, such as the relative index of inequality [RII], which takes into account the whole socioeconomic distribution were also extracted.^11^ To enhance comparability between studies, all ratios were re-calculated so that the highest socioeconomic category was compared with the lowest and survival ratios were transformed to fatality ratios. Additional *P* values or confidence intervals were calculated when missing.

Meta-analyses were conducted separately for studies of incidence, case fatality and mortality. The relative measures (i.e. HR, RR, OR and SIR) from the cohort studies in each section were stratified into subgroups based on the covariates in the corresponding regressions to control for potential confounding factors. Case-control studies and studies using RII differ methodologically from the other studies and were not included in the meta-analysis. If regressions contained multiple SES measures only one measure was included, where education was prioritised over income and income was prioritised over occupation. Summary relative risks (SRRs) were estimated for each subgroup containing two or more studies using a random effects model (results in subsamples within studies were first pooled using the same method). To test for heterogeneity between studies, the I^2^-statistic was used. Funnel plots, where the relative risks in the individual studies are plotted against the standard errors, were used to graphically examine small-study effects.^12^ In the absence of small-study effects, studies with large standard errors will scatter widely at the bottom of the graph around the SRR illustrated by a vertical line. Additionally, the Egger’s test was used to formally test for small-study effects in the meta-analysis. All statistical analyses were performed using STATA version 13.0.^13^

## Results

**Figure 1:**
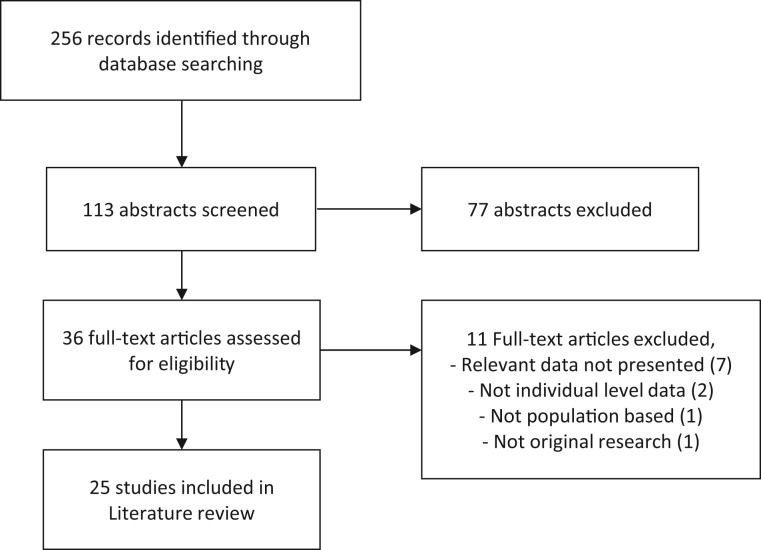
PRISMA flow diagram of the study selection process

We selected 113 articles for review of abstracts, of which 36 were selected for full-text review. Of the 36 full-text articles, 25 fulfilled the eligibility criteria and were included in the review, and 11 were excluded as they did not present relevant data (*n* = 7), lacked individual-level data (*n* = 2), were not population based (*n* = 1), or did not involve original research (*n* = 1). A flow diagram of the study selection process is presented in figure 1.

Of the 25 included articles, 7 studied incidence only,^14–20^ 9 studied case fatality only,^21–29^ 8 studied mortality only,^30–37^ while one studied both incidence and case fatality.^38^ None of the articles studied all three outcome measures together. Five studies were conducted in Sweden,^14^^,^^24^^,^^26^^,^^37^^,^^38^ five in Denmark,^15–17^^,^^23^^,^^27^ four in France,^20^^,^^25^^,^^32^^,^^33^ two in Norway,^30^^,^^36^ two in the Netherlands^21^^,^^29^ and one each in Italy,^18^ Iceland,^19^ Belgium,^31^ Switzerland,^22^ the UK^34^ and Ireland^28^ while one study combined data from eleven European regions.^35^ In total, 12 studies used national register data, 8 regional register data, 2 demographic sample data, 1 multinational register data and 2 hospital interview data. The majority of the studies had a cohort design, while two were case-control studies.^18^^,^^20^ Detailed descriptions of the articles are presented in tables 1–3. All studies scored high (between 6 and 9 stars) on the Newcastle-Ottawa quality assessment scale, (details found in Supplementary table S2 in the Supplementary material).

**Table 1:** Characteristics of studies with breast cancer incidence as an outcome

Author, year (location)	Incidence measure	Socioeconomic measure (number of groups)	Covariates	Population	Data source	Study design	Statistical method
Meijer et al.^17^, 2013 (Denmark)	Not specified	Education (3), household income (4), occupation (10)	Age, invitation to mammography screening, marital status, residential factors	1 539 162 women	Statistics Denmark, National Cancer Register	Cohort study	Shared frailty model
Beiki et al.^14^, 2012 (Sweden)	Invasive	Education (3)	Age, ethnicity, parent’s ethnicity	4 553 484 women	National Cancer Registry, LISA database, Multi-Generation Register, Cause of Death Register	Cohort study	Poisson model
Petracci et al.^18^, 2011 (Italy)	Invasive	Education (3)	Age, family history of breast cancer, previous breast biopsies, age at first birth, age at menarche, physical activity, alcohol, BMI	2523 women with breast cancer and 2504 control subjects	Structured interviews at major hospitals in six Italian regions	Case-control study	Logistic model
Villeneuve et al.^20^, 2011 (France)	Invasive and *in situ* combined	Education (4)	Age, study area, hormone replacement therapy, family history of breast cancer, history of benign breast disease, parity, age at first birth, duration of breastfeeding, age at menarche, BMI	1230 women with breast cancer and 1315 control subjects	Structured interviews at hospitals in two French regions	Case-control study	Logistic model
Larsen et al.^16^, 2011 (Denmark)	Not specified	Education (3), income (4), occupation (7)	Age, hormone replacement therapy, parity, age at first birth, alcohol, BMI	23 111 women	National Cancer Registry, IDA database, Diet, Cancer & Health cohort study	Cohort study	Cox proportional hazard model
Carlsen et al.^15^, 2008 (Denmark)	Invasive	Education (3), household income (3), occupation (6)	Age, time period	1 589 789 women	National Cancer Register, National Patient Register, IDA database, Central Population Register	Cohort study	Poisson regression
Vidarsdottir et al.^19^, 2008 (Iceland)	Not specified	Education (3)	Age, time period	58 505 women	The 1981 Census, National Cancer Registry	Cohort study	SIR
Hussain et al.^38^, 2008 (Sweden)	Invasive and *in situ* separately	Education (4)	Age, time period, family history of breast cancer, parity, age at first birth, residential factors	1 571 511 women	National Family-Cancer Database, Cause of Death Register	Cohort study	Cox proportional hazard model

**Table 2:** Characteristics of studies with case fatality as an outcome

Author, year (location)	Case fatality measure	Socioeconomic measure (number of groups)	Covariates	Population	Data source	Study design	Statistical method
Larsen et al.^27^, 2015 (Denmark)	All-cause	Education (3), income (3)	Age, tumour size, lymph nodes, grade, receptor status, comorbidity, BMI, diabetes, smoking status, alcohol intake	1227 postmenopausal women primary breast cancer	National Cancer Registry, National Patients Registry, IDA database, Diet, Cancer & Health cohort study	Cohort study	Cox proportional hazards regression
Walsh et al. ^28^, 2014 (Ireland)	Cancer-specific	Area-based index (5)	Age, time period, TNM-stage, tumour size, grade, morphology, receptor status, method of presentation, surgery, radiotherapy, chemotherapy, hormone therapy, comorbidity, smoking status, residential factors	19 694 women with invasive breast cancer	National Cancer Registry, Cause of Death registry	Cohort study	Poisson regression & Cox proportional hazards regression
Aarts et al.^29^, 2011 (Netherlands)	Cancer-specific	Area-based income (3)	Age, stage, surgery, radiotherapy, systemic therapy, comorbidity	5331 women with invasive breast cancer	Regional Cancer Registry, Screening program, Statistics Netherlands	Cohort study	Cox proportional hazards regression
Bastiaannet et al.^21^, 2011 (Netherlands)	All-cause	Area-based index (5)	Age, TNM-stage, histology, grade, surgery, adjuvant treatment	127 599 women with primary breast cancer (invasive or *in situ*)	National Cancer Registry, Netherlands Institute for Social Research	Cohort study	Cox proportional hazards regression
Eaker et al.^24^, 2009 (Sweden)	Cancer-specific	Education (3), income (2), household income (2), occupation (2) household occupation (2)	Age, time period, tumour stage, tumour size, lymph nodes, proliferation status, receptor status, surgery, radiation, chemotherapy, hormonal therapy	9908 women with primary invasive breast cancer	Regional Breast Cancer Registry, LISA database, Multi-Generation Register, Cause of Death Register	Cohort study	Cox proportional hazards regression
Gentil-Brevet et al.^25^, 2008 (France)	All-cause	Occupation (2)	Age, tumour stage, history of mammography, cancer detected by screening mammography, parity, marital status, residential factors	1138 women with invasive breast cancer	Five Regional Cancer Registers	Cohort study	Cox proportional hazards regression model
Hussain et al.^38^, 2008 (Sweden)	Cancer-specific	Education (4)	Age, time period, family history of breast cancer, parity, age at first birth, residential factors	43 222 women with primary invasive breast cancer	National Family-Cancer Database, Cause of Death Register	Cohort study	Cox proportional hazards regression model
Dalton et al.^23^, 2007 (Denmark)	Cancer-specific & All-cause l	Education (3), household income (4), occupation (6)	Age, tumour size, lymph nodes, histologic grade and type, receptor status, comorbidity, residential factors	25 897 women with primary invasive breast cancer	National Breast Cancer Register, National Patient Registry, IDA database, Cause of Death register	Cohort study	Cox proportional hazards regression model
Bouchardy et al.^22^, 2006 (Switzerland)	Cancer-specific	Occupation (4)	Age, tumour stage, tumour size, lymph nodes, histologic type, receptor status, differentiation, method of detection, surgery, radiotherapy, chemotherapy, hormonal therapy, marital status, country of birth	3920 women with invasive breast cancer	Regional Cancer Registry, Cantonal Population Office	Cohort study	Cox proportional hazards regression model
Lagerlund et al.^26^, 2005 (Sweden)	Cancer-specific	Education (3), income (4), household income (4), occupation (2), household occupation (2)	Age, tumour size, lymph nodes, parity, residential factors	4645 women with first invasive breast cancer	National Cancer Register, five Regional Cancer Registers, Population and Housing Census, Fertility Register, Migration Register, Cause of Death Register	Cohort study	Cox proportional hazards regression model

**Table 3:** Characteristics of studies with breast cancer mortality as an outcome

Author, year (location)	Socioeconomic measure	Covariates	Population	Data source	Study design	Statistical method
Menvielle et al.^33^, 2013 (France)	Education (5)	Age, time period	130 980 women in 1990–1998 and 137 833 in 1999–2007	Echantillon Démographique Permanent, Cause of Death Register	Cohort study	RII (Cox).
Gadeyne et al.^31^, 2012 (Belgium)	Education (4)	Age, parity, age a first birth	2 247 699 women	The 1991 Census, Cause of Death Register	Cohort study	Poisson model
Elstad et al.^30^, 2012 (Norway)	Education (3)	Age, time period	All Norwegian women aged 45–74 sometime during 1971–2002 (circa 21 million person-years)	Linking of National Registers by Statistics Norway	Cohort study	Logistic Model & RII (logistic)
Weires et al.^37^, 2008 (Sweden)	Occupation (9)	Age, time period, parity, age at first birth, residential factors	1 025 856 women	The Swedish Family-Cancer Database, Cause of Death Register	Cohort study	Cox proportional hazard model
Strand et al.^35^, 2007 (Finland, Norway, Denmark, England and Wales, Belgium, France, Switzerland, Austria, Turin, Barcelona and Madrid)	Education (3)	Age, marital status	1 296 959 women in Finland, 987 441 in Norway, 1 274 530 in Denmark, 129 074 in UK, 2 530 405 in Belgium, 123 237 in France, 1 957 865 in Austria, 1 096 329 in Switzerland, 265 095 in Turin, 437 104 in Barcelona and 1 251 541 in Madrid	Longitudinal mortality data from Finland, Norway, Denmark, England and Wales, Belgium, France, Switzerland, Austria, Turin, Barcelona and Madrid for participants in the early 1990s Censuses	Cohort study	RII (Poisson)
Menvielle et al.^32^, 2006 (France)	Education (4)	Age, time period	94 734 women in 1968, 99 737 in 1975, 100 898 in 1982 and 112 066 in 1990	Echantillon Démographique Permanent, Cause of Death Register	Cohort study	RII (Cox).
Strand et al.^36^ 2005 (Norway)	Education (3)	Age, parity, age at first birth	528 517 women	The 1990 Census, Cause of Death Register	Cohort study	Cox proportional hazard model
Power et al.^34^, 2005 (UK)	Occupation (4)	Age, BMI, smoking, father’s social class	11 855 women	Perinatal Mortality Survey, National Health Service Central Register	Cohort study	Cox proportional hazard model

SES was measured by education in 18 studies,^14–20^^,^^23^^,^^24^^,^^26^^,^^27^^,^^30–33^^,^^35^^,^^36^^,^^38^ while 8 used income,^15–17^^,^^23^^,^^24^^,^^26^^,^^27^^,^^29^ 10 occupation^15–17^^,^^22–26^^,^^34^^,^^37^ and 2 socioeconomic indices.^21^^,^^28^ About half of the studies only included one socioeconomic variable while the other half included several variables. The majority of the studies used individual or household level socioeconomic variables, but three used area-based measurements.^21^^,^^28^^,^^29^ Education was measured as the highest attained education of the woman and was categorised into three to five levels. Income was based on either individual or household disposable income and divided into two to four groups. The number of occupational categories ranged from two to 10 and was measured by the occupation of the woman or her husband.

A number of methods were used to estimate relative risk ratios. Cox proportional hazard regression models were used to estimate HR in all studies of case fatality and some studies of incidence and mortality. Some studies of incidence and mortality used Poisson regression models to estimate RR. The case-control studies used logistic regression models to estimate OR. The majority of the studies made pairwise comparisons between socioeconomic groups, generally the highest and lowest. Four studies of mortality used the relative inequality index, based on Cox regression,^32^^,^^33^ Poisson regression^35^ or logistic regression.^30^ Detailed results from the studies are presented in Supplementary tables S3–S5 in the Supplementary material. The funnel plots and the Egger’s test for funnel plot asymmetry are presented in Supplementary figure S1 and table S6 in the Supplementary material.

### Incidence

Eight studies examined the association between breast cancer incidence and education,^14–20^^,^^38^ income^15–17^ or occupation^15–17^ (table 1). Incidence was either measured by invasive cancer,^14^^,^^15^^,^^18^ invasive and *in situ* cancer combined,^20^ invasive and *in situ* cancer separately^38^ and was in three studies not specified.^16^^,^^17^^,^^19^

**Figure 2:**
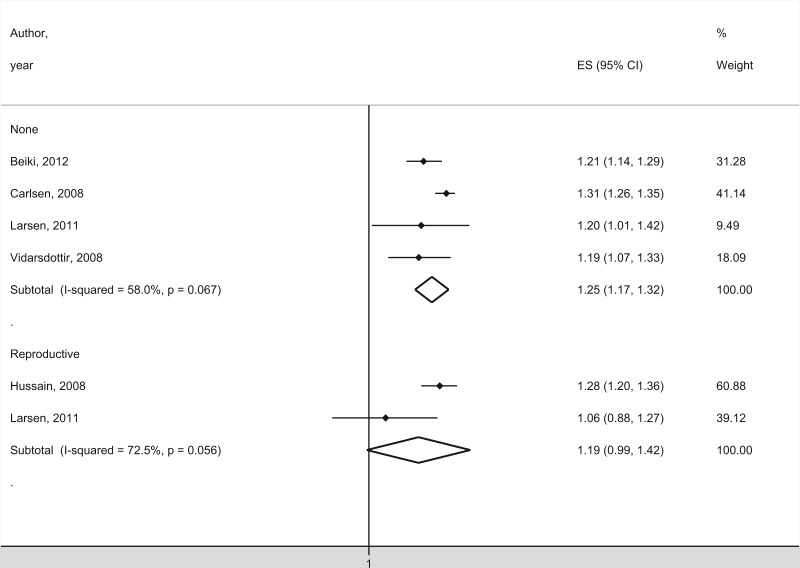
Meta-analysis of studies with incidence as outcome in relation to SES. The studies are organised by included covariates. The black squares and horizontal lines correspond to the study-specific relative risks and 95% confidence intervals, while the diamonds represent the pooled relative risk and the 95% confidence interval

The five studies that controlled for age or calendar period reported significantly positive associations between breast cancer incidence and education,^14–16^^,^^19^^,^^20^ income^15^ or occupation.^15^^,^^16^ Increased incidence was found for highest vs. lowest education,^14–16^^,^^19^^,^^20^ highest vs. middle income,^15^ skilled vs. unskilled workers^16^ and creative core vs. manual occupation.^15^ One study found no significant association for income after controlling for education and occupation,^16^ while another study reported weakened but significant associations for occupation after controlling for education and income.^15^ The meta-analysis included four studies and showed a significantly higher incidence of breast cancer in women with high SES (figure 2) with a SRR of 1.25 (1.17–1.32). Although the funnel plot indicated possible small-study effects the Egger’s test for small-study effects was not significant (*P* = 0.20).

Four studies controlled for reproductive factors,^16^^,^^18^^,^^20^^,^^38^ hormone replacement therapy,^16^^,^^20^ family history of breast cancer^18^^,^^20^^,^^38^ and lifestyle factors^16^^,^^18^^,^^20^ in addition to age and time period. Women with a high SES had significantly higher age at first birth and a lower parity.^38^ Breast cancer incidence was significantly higher among women with a higher age at first birth,^18^^,^^20^ low parity,^20^ low age at menarche,^20^ use of hormone replacement therapy,^20^ family history of breast cancer,^18^ BMI^18^^,^^20^ and alcohol intake.^18^ When controlling for reproductive factors and other covariates, a significantly higher incidence was found for women with higher education.^18^^,^^38^ There also seemed to be a stronger association between education and *in situ* cancer compared with invasive cancer. For invasive cancer, the association remained significant for both ductal and lobular cancer as well as for women aged 30–49 and 50–64 years.^38^ One study no longer found an association between incidence and education after controlling for reproductive factors.^20^ The meta-analysis contained two studies that controlled for reproductive factors and did not show a significant association between incidence and SES (figure 2) with a SRR of 1.19 (0.99–1.42, *P* = 0.06). Although the funnel plot indicated possible small-study effects no Egger’s test was possible due to the small number of studies included in the meta-analysis.

One study controlled for mammography screening by including information about invitation to breast cancer screening program.^17^ Women invited to screening within two years had a significantly higher breast cancer incidence compared with women who had not been invited to screening. Controlling for screening in addition to age and other covariates, there was a significant positive association between breast cancer incidence and education, HR = 1.08 (1.03–1.14), as well as with income and occupation.^17^ The meta-analysis on breast cancer incidence did not include studies controlling for mammography screening since there was only one such study found in the literature review.

### Case fatality

Ten studies investigated the association between case fatality and education,^23^^,^^24^^,^^26^^,^^27^^,^^38^ income,^23^^,^^24^^,^^26^^,^^27^^,^^29^ occupation,^22–26^ or a socioeconomic index^21^^,^^28^ (table 2). Six studies measured case fatality by cancer-specific fatality,^22^^,^^24^^,^^26^^,^^28^^,^^29^^,^^38^ three by all-cause fatality^21^^,^^25^^,^^27^ while one study used both.^23^

**Figure 3:**
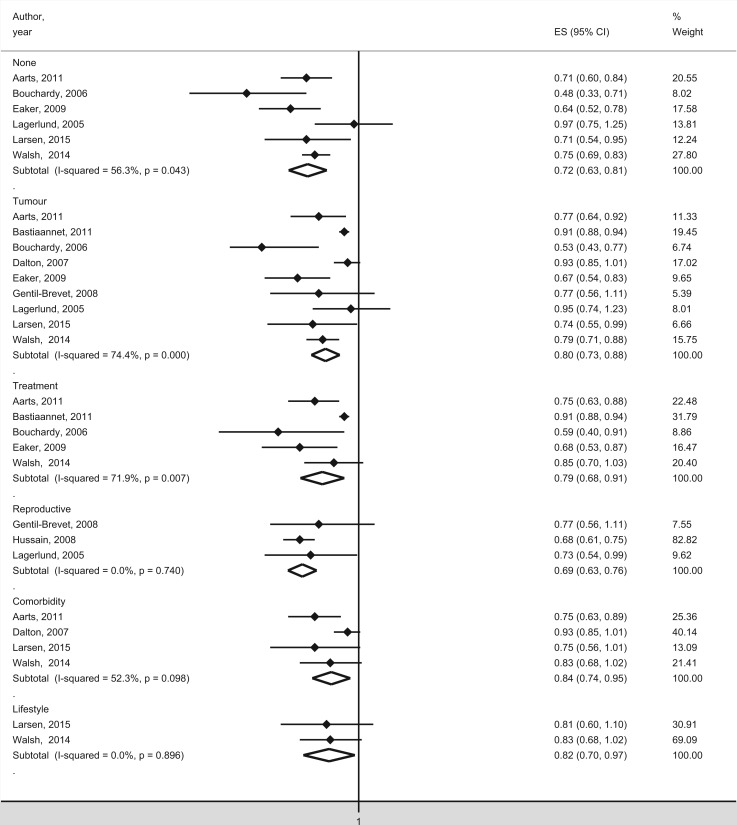
Meta-analysis of studies with case fatality as outcome measure in relation to SES. The studies are organised by included covariates. The black squares and horizontal lines correspond to the study-specific relative risks and 95% confidence intervals, while the diamonds represent the pooled relative risk and the 95% confidence interval

All six studies that solely controlled for age (and sometimes time period) identified a significant, inverse association between case fatality and education,^24^^,^^27^ income,^26^^,^^29^ occupation^22^^,^^26^ or socioeconomic index.^28^ Significantly lower case fatality was found for women with high education, income and occupational group. One study reported a continuous significant negative association during 1994–1998, 1999–2003 and 2004–2008^28^ between case fatality and socioeconomic index. One study showed a negative significant association between case fatality and income for screen-detected cancers, interval cancers and cancer in non-attenders,^29^ while another study showed a negative significant association with socioeconomic index for symptomatic cancers but not for screen-detected cancers.^28^ A negative significant association between case fatality and education was found for women aged 65 years or younger^24^ and with occupation in women younger than 50 years.^26^ The meta-analysis showed a significantly lower case fatality for women with high SES (figure 3), with a SRR of 0.72 (0.63–0.81). Although the funnel plot suggested possible small-study effects the Egger’s test for small-study effects was not significant (*P* = 0.53).

Eight studies included tumour stage^22^^,^^24^^,^^25^^,^^28^^,^^29^ or tumour size^23^^,^^26–28^ as covariates and some also included other tumour characteristics, such as histology, grade or receptor status. A low SES was significantly correlated with a more advanced stage at diagnosis^21^^,^^22^^,^^24^^,^^25^^,^^28^^,^^29^ and a higher stage was associated with higher case fatality.^21^^,^^23–26^ Seven studies including tumour stage or size found a negative significant association between case fatality and education,^24^^,^^27^ income,^23^^,^^24^^,^^29^ occupation^22^^,^^24^^,^^26^ or socioeconomic index,^28^ while one study found no significant association.^25^ In one study, education remained significant for stages I, IIa, III–IV but not for stage IIb.^24^ In one study, occupation remained significant after including method of detection,^22^ while in another study income remained significant for screen-detected cancers but not for interval cancers, or for non-attenders.^29^ The meta-analysis of studies controlling for tumour characteristics yielded a SRR of 0.80 (0.73–0.88) indicating a significantly lower case fatality for women with high SES (figure 3). However, the funnel plot and the Egger’s test indicated small-study effects (*P* values = 0.02).

Only one study included participation in mammography screening as a covariate,^25^ while another study stratified results by method of detection.^28^ Attendance in mammography screening was significantly higher for women with high SES.^28^ Mammography screening was significantly associated with lower case fatality, also after adjustment of tumour stage. There was a significant negative association between case fatality and occupation when controlling for mammography screening, HR = 1.40 (1.00–1.90), but not after further adjustment for tumour characteristics, HR = 1.30 (0.90–1.80, *P* = 0.11).^25^

All five studies that adjusted for treatment factors found a negative association between case fatality and SES.^21^^,^^22^^,^^24^^,^^28^^,^^29^ Breast-conserving surgery was more often performed in women with high SES.^22^^,^^24^^,^^28^ One study found that radiation therapy after breast-conserving surgery was more common in women with high SES,^24^ while two studies did not found such association.^22^^,^^28^ In one study, income remained significant for interval cancers and non-attenders but not for screening-detected breast cancers.^29^ In the other four studies, there was a negative significant association between case fatality and SES after adjusting for treatment factors in combination with tumour characteristics.^21^^,^^22^^,^^24^^,^^28^ The meta-analysis of studies controlling for treatment factors indicated a significantly lower case fatality for women with high SES (figure 3), with a SRR of 0.79 (0.68–0.91). However, the funnel plot and the Egger’s test suggested small-study effects (*P* values = 0.02).

Three studies included reproductive factors in the form of parity^25^^,^^26^^,^^38^ or age at first birth.^38^ A high SES was significantly correlated with higher age at first birth and lower parity^38^ and low parity was significantly associated with higher case fatality.^25^ A negative significant association between case fatality and SES remained after inclusion of reproductive factors alone^38^ or in combination with tumour characteristics.^25^^,^^26^^,^^38^ The meta-analysis of studies controlling for reproductive factors resulted in a SRR of 0.69 (0.63–0.76) revealing a significantly lower case fatality for women with high SES (figure 3).

Four studies controlled for comorbidity.^23^^,^^27–29^ A high SES was significantly associated with a lower degree of comorbidity^23^^,^^27^^,^^28^ and comorbidity was significantly associated with higher case fatality.^23^ A negative significant association between case fatality and income remained for interval cancers and screening non-attenders after controlling for comorbidity.^29^ After adjusting for comorbidity in combination with tumour characteristics, one study reported a significant negative association between case fatality and income^23^ while another study found no significant association for education or income.^27^ No significant association was found after including comorbidity, tumour characteristics and treatment factors at the same time.^29^ One study found a negative significant association between case fatality and socioeconomic index for patients without comorbidity but not for patients with comorbidity.^28^ The meta-analysis of studies controlling for comorbidity showed a significantly lower case fatality for women with high SES (figure 3), with a SRR of 0.84 (0.74–0.95). Although the funnel plot indicated possible small-study effects the Egger’s test for small-study effects was not significant (*P* = 0.13).

Two recent studies included lifestyle factors in addition to tumour characteristics.^27^^,^^28^ Smoking and obesity were significantly associated with higher case fatality^27^ and both factors were significantly less common among women with high SES.^27^^,^^28^ No significant association was found in the study that only controlled for smoking^28^ or the study that included smoking, obesity and alcohol intake.^27^ The meta-analysis of studies that controlled for lifestyle factors indicated a significantly lower case fatality for women with high SES (figure 3) with a SRR of 0.82 (0.70–0.97).

### Mortality

**Figure 4:**
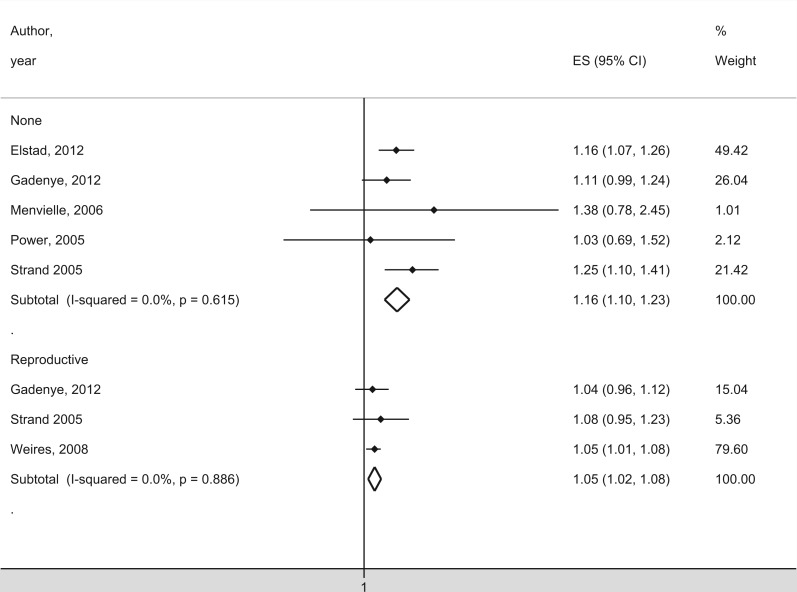
Meta-analysis of studies with mortality as outcome measure in relation to SES. The studies are organised by included covariates. The black squares and horizontal lines correspond to the study-specific relative risks and 95% confidence intervals, while the diamonds represent the pooled relative risk and the 95% confidence interval

Eight studies examined the association between breast cancer mortality and education^30–33^^,^^35^^,^^36^ or occupation^34^^,^^37^ (table 3). Studies on the influence from education on breast cancer mortality reached contradictory results with a significant positive association in three studies^31^^,^^35^^,^^36^ and a significant negative association in two studies,^30^^,^^32^ while a follow-up study found no significant association with education.^33^ Another study found no significant association between occupation and mortality.^34^ Two studies reported a positive significant association for women 50 years or older^31^^,^^35^ but not for women below the age of 50. In two other studies, the association changed over time; one study found no significant association in 1971–1979, but found a significant positive association in 1980–2002,^30^ while the other study found a significant positive association during 1968–1981 but not during 1990–2007.^32^^,^^33^ One study also found a significant positive association for married woman but not for women who never married.^35^ The meta-analysis included five studies and demonstrated a significantly higher mortality for women with high SES (figure 4), with a SRR of 1.16 (1.10–1.23).

Three studies controlled for reproductive factors.^31^^,^^36^^,^^37^ A significantly higher mortality was associated with high age at first birth and low parity.^31^^,^^36^ Two studies found a significant positive association between breast cancer mortality and SES when controlling for at least one reproductive factor at a time.^36^^,^^37^ One study observed a positive significant association between education and mortality when controlling for parity but not when controlling for age at first birth.^36^ When controlling for parity and age at first birth at the same time, one study found a positive association between mortality and occupation,^37^ while two studies found no significant association between mortality and education.^31^^,^^36^ One study found a positive significant association between education and mortality among nulliparous women,^36^ while another found no significant association.^31^ The meta-analysis of studies controlling for reproductive factors indicated a significantly higher mortality for women with high SES (figure 4), with a SRR of 1.05 (1.02–1.08).

## Discussion

This systematic review and meta-analysis with focus on SES-related inequalities in breast cancer demonstrate a significantly higher incidence of breast cancer in women with higher SES when controlling for age and time period. After adding reproductive factors as an explaining factor, the association between higher incidence and SES becomes non-significant in the meta-analysis (*P* = 0.06). One study controlled for invitation to screening (not included in the meta-analysis) and found that the increased incidence for women with higher SES could partly be explained by screening attendance.^17^

Case fatality was significantly lower in women with a higher SES. Tumour characteristics, treatment factors, comorbidity and lifestyle factors seem to partly explain the lower case fatality, which was not influenced by reproductive factors. Stage at diagnosis influenced case fatality for interval cancers and non-screening diagnosed tumours, whereas treatment factors and comorbidity are estimated to represent the major reasons for case fatality in screening-detected cancers.^29^ Our meta-analysis shows a significant positive association between breast cancer mortality and SES, which to some extent was explained by reproductive factors. Data on for example tumour characteristics, treatment, comorbidity and lifestyle factors, which have been correlated with SES and case fatality, were not available.

A limitation in the meta-analyses is a potential bias arising from small-study effects, as shown by some of the funnel plots. However, only two of the meta-analyses on studies on case fatality indicated significant small-study effects according to the Egger’s test. This does not alter the interpretation of the results since we still expect the SRR to be significantly different from zero, although the effect might be smaller than indicated by the meta-analyses.

The quality of the included studies was rated high according to the Newcastle-Ottawa quality assessment scale. A restriction in several studies was, however, the lack of information on relevant covariates. Although mammography screening is known to decrease breast cancer mortality and increase incidence,^39^ only one study of breast cancer incidence and two studies of case fatality contained information on mammography screening. Other known risk factors include use of hormone replacement therapy and oral contraceptives.^6^ Data on hormone therapy were available in two studies of breast cancer incidence, whereas data on oral contraceptives were missing. The studies on which the meta-analyses were based showed significant heterogeneity related to SES, statistical methods, countries, time periods and sample sizes. Also, SES measures differ in types and categories and in their classification of occupations, which complicate cross-study comparison. Further, standardised relative outcome measures and greater power would improve the validity of meta-analyses.

A recent meta-analysis focused on breast cancer risk and mortality linked to residential area also found a significantly higher incidence for women with high SES, but could not demonstrate an association between mortality and SES.^7^ This review excluded studies that lacked area-based measures, which led to overweight for US-based studies with inclusion of only four European studies, which have the advantage of a population-basis and individual-level data that positively contributes to data validity.

The higher incidence of breast cancer in women with high SES is predominantly linked to reproductive factors and to a lesser degree by the use of hormone replacement therapy. Both of which are efforts, unless the reproductive factors are affected by infertility, in which case it is considered as a circumstance. Due to difficulties in identifying the influence of circumstance on effort, both factors may be considered as illegitimate. In this regard, hormone replacement therapy seems particularly relevant to target by policy intervention with the aim to reduce socioeconomic inequalities in breast cancer incidence.

The lower case fatality in women with higher SES seems to be explained by both circumstances, i.e. treatment factors, and efforts, i.e. comorbidities and lifestyle factors. It is also explained by stage at diagnosis, which is influenced by tumour aggressiveness (circumstance) and participation in mammography screening (effort). Participation in mammography screening represents a suitable target for policy interventions to reduce socioeconomic inequalities in breast cancer fatality. Comorbidity and lifestyle factors are efforts that might be harder to affect by policy intervention, but that warrant further in-depth analysis regarding their influence on diagnostics, treatment and follow-up for breast cancer.

Unexplained socioeconomic inequalities in breast cancer incidence, case fatality and mortality remain even after controlling for reproductive factors, use of hormone replacement therapy, stage at diagnosis, treatment factors, comorbidities and life-style factors. To gain further insight into the complex inter-relation between these factors, simultaneous inclusion of breast cancer incidence, case fatality and mortality as outcomes and inclusion of all relevant covariates, such as screening attendance, use of contraceptives, lifestyle and reproductive factors could contribute to a better understanding of socioeconomic inequalities in breast cancer.

## Funding

This work was supported by a Government Grant for Clinical Research (‘ALF’).

## Supplementary data

Supplementary data are available at *EURPUB* online.

*Conflicts of interest*: None declared.

Key points

- A higher SES is significantly associated with higher breast cancer incidence, lower breast cancer case fatality and higher breast cancer mortality.
- Participation in mammography screening is a suitable target for policy interventions to reduce socioeconomic-related inequalities in breast cancer.
- Comorbidity and lifestyle factors are difficult to affect by policy intervention but warrants further in-depth analysis regarding their influence on breast cancer treatment.
